# Structure of the human nonmuscle myosin 2A motor domain: Insights into isoform-specific mechanochemistry

**DOI:** 10.1016/j.jbc.2025.110691

**Published:** 2025-09-10

**Authors:** Robin S. Heiringhoff, Johannes N. Greve, Michael Zahn, Dietmar J. Manstein

**Affiliations:** 1 Institute for Biophysical Chemistry, Fritz–Hartmann–Centre for Medical Research, Hannover Medical School, Hannover, Germany; 2 Division for Structural Biochemistry, Hannover Medical School, Hannover, Germany

**Keywords:** myosin, actin, molecular motor, crystal structure, molecular dynamics, nonmuscle myosin-2, actomyosin interactions, isoform-specific functions

## Abstract

Nonmuscle myosin 2A (NM2A) is the predominant myosin isoform in nonmuscle cells. Together with its paralogues NM2B and NM2C, NM2A enables tension and force generation, driving essential cellular processes such as membrane protrusion and retraction, directed migration, adhesion, and cytokinesis. The NM2 isoforms display paralogue-specific mechanochemical characteristics that support their specific cellular functions. Here, we determined the structure of the human NM2A motor domain, addressing a critical gap in understanding myosin family diversification. Based on our experimentally resolved 2.1 Å structure of the NM2A motor domain in its nucleotide-free state, we demonstrate, through integrative modeling of NM2-actin complexes and molecular dynamics simulations, how sequence differences between NM2A and NM2B underpin their functional specialization. Loop2 emerges as a critical determinant of isoform-specific behavior. Comparative analysis of ATP interaction fingerprints across NM2 isoforms reveals a conserved ATP binding mechanism. These findings illuminate an allosteric energy transduction pathway that connects sequence variation to actin-binding dynamics, providing mechanistic insight into isoform-specific cytoskeletal functions.

Myosins are molecular motors that convert ATP-derived chemical energy into mechanical force. The nonmuscle myosin 2 (NM2) paralogues—NM2A, NM2B, and NM2C—are essential for various cellular processes, including cytokinesis, cell migration, cytoskeletal organization, endocytosis, exocytosis, vesicle trafficking, and membrane remodeling. The heavy chains of NM2 isoforms are encoded by *MYH9*, *MYH10*, and *MYH14*, respectively. Functional NM2 complexes are hexameric, consisting of two heavy chains, two essential light chains encoded by *MYL6*, and two regulatory light chains (RLCs), which are expressed in a tissue-type-specific and cell-type-specific manner by *MYL9*, *MYL12A*, or *MYL12B* (1, 2). The function of NM2 is regulated by the phosphorylation of its RLC at a specific serine residue within the target sequence PQRATSNVF, located near the N terminus of each of the three alternative RLCs. Phosphorylation induces conformational changes, triggering the unfolding of the NM2 hexamer. RLC phosphorylation by MLCKs or ROCKs disrupts intramolecular interactions that stabilize the folded, inactive conformation and shifts the equilibrium to extended conformations, promoting the assembly of NM2 into bipolar filaments (3, 4). Concurrently, the motor domains undock, enabling actin binding, nucleotide exchange, and the resumption of the mechanochemical cycle. Each NM2 paralogue has distinct tissue distribution and functional roles, although they often overlap. NM2A is typically the most abundant paralogue in many nonmuscle cell types, and is widely produced, with roles in generating strong contractile forces, cell adhesion, and maintaining cortical tension (5). Its broad distribution makes it crucial for basic mechanical functions in most cells (6). NM2B is less abundant than NM2A in many cell types, but is still present at significant levels (7, 8). NM2B has slower kinetics and is important for processes requiring sustained force generation, such as cell polarity and supporting prolonged structural changes in various cell types (9, 10, 11). Higher levels of NM2B are often found in specialized tissues like neuronal and cardiac cells (12, 13, 14). NM2C, by far the least abundant paralogue overall, is produced in multiple tissues and has an important function in epithelial and neuronal tissue by regulating microvilli length and neuronal process outgrowth (15, 16, 17).

The expression of the genes encoding NM2 paralogues is tightly regulated, ensuring precise control over their levels and distribution to meet specific cellular and tissue requirements. Fibroblast, blood cells, and endothelial cells typically exhibit high levels of NM2A, which facilitates tension generation and cell adhesion. In contrast, neuronal cells have higher NM2B levels to ensure sustained contractile activity essential for processes such as axon guidance (12, 13, 18, 19). Secretory cells, on the other hand, rely on NM2C for spreading and vesicle dynamics (16, 20). This variability in abundance ensures that cells can fine-tune their contractile machinery for specific physiological functions (1). Mutations in *MYH9* affecting the motor domain have been shown to cause an autosomal dominant disorder. *MYH9*-related disease (*MYH9*-RD) is characterized by thrombocytopenia, giant platelets and Döhle body-like inclusions in peripheral blood leukocytes with variable ultrastructural features. Furthermore, some patients exhibit symptoms consistent with Alport syndrome, including nephritis, hearing loss, and ocular abnormalities (21, 22).

Each NM2 heavy chain is composed of three distinct domains: the motor domain, the neck domain, and the tail domain. The motor domain, which drives ATP hydrolysis, is composed of the N-terminal Src homology 3 (SH3)-like subdomain, the upper 50 kDa domain (U50), the lower 50 kDa domain (L50), and the converter (23, 24). A large cleft between the U50 and L50 domains bridges the active site within the motor domain and the actin-binding region. This region is composed of five flexible surface loops (loop2, CM-loop, loop4, activation loop, and loop3), along with a helix-loop-helix motif. These structural elements form a dynamic surface that facilitates actin interaction, with conformational changes in the cleft driving the modulation of actin binding. These conformational changes are nucleotide-dependent, driven by the ATP-bound state within the pocket (25, 26). The converter domain has been found to amplify these changes and transmit them to the lever arm, composed of the neck domain and associated light chains, thereby enabling force generation and actin filament sliding (26).

NM2A, NM2B, and NM2C exhibit distinct mechanochemical properties (1). NM2A shows the highest ATPase activity and moves actin filaments the fastest *in vitro*, compared to NM2B and NM2C. Further paralogue-specific differences are observed in the duty ratio of the motor, defined as the proportion of the ATPase cycle during which the myosin motor is associated with the actin filament. In the absence of external load and any other modulating factor, NM2A displays the lowest duty ratio compared to NM2B and NM2C (9, 27, 28). A diverse array of tropomyosin isoforms is produced by cells, and their selective association with actin filaments play a role in fine-tuning myosin motor activity (29). Processive movement of individual NM2A and NM2B molecules was observed along actin filaments decorated with different tropomyosin isoforms. *In vitro* motility assays have been used to show that both isoform identity and N-terminal acetylation modulate the duty ratio, affecting the kinetics and chemomechanical properties of NM2A and NM2B under loaded and unloaded conditions (30, 31, 32).

To gain deeper insight into the structural changes associated with ATP hydrolysis, myosin structures have been extensively studied in various nucleotide states using ATP analogs (33, 34, 35). Crystal structures of the NM2B and NM2C motor domains have been resolved in the nucleotide-free and ADP•VO_4_ bound state, respectively (36, 37). However, until now, the structure of the NM2A motor domain had not been determined. Here, we present the crystal structure of the NM2A motor domain fused to an artificial lever arm in its nucleotide-free state, resolved to a resolution of up to 2.0 Å. Comparative analysis with NM2B and NM2C reveals a high degree of structural conservation across the subdomains of the myosin motor. Moreover, integrative modeling combined with molecular dynamics simulation provides valuable insights into the dynamics and conformational flexibility of the actin-binding loop regions.

## Results and discussion

### Structure of the NM2A motor domain

The fusion construct, comprising the NM2A motor domain linked to the artificial lever arm, was produced in *Spodoptera frugiperda* (Sf9) cells, purified and used for crystal setups with commercial screens. Multiple conditions produced crystals, with one condition further optimized to the final condition consisting of 16% PEG3350 and 240 mM Na-thiocyanate. The resulting crystal belonged to the space group P2_1_2_1_2_1_ and diffracted to nearly 2.0 Å resolution (Table 1). Due to the anisotropic nature of the diffraction data, it was processed using STARANISO. The structure was subsequently solved by molecular replacement and refined, resulting in a model with R_work_ and R_free_ values of 21.97% and 26.07%, respectively (Table 1).

**Table 1:** Crystallographic data, phasing, and refinement statistics

Protein	NM2A-2R
PDB–code	9IHL
Data collection	
Beamline	DLS I03
Space group	P2_1_2_1_2_1_
Cell parameters: *a*, *b*, *c* [Å] α, β, γ [°]	61.0, 120.9, 149.4 90, 90, 90
Wavelength [Å]	0.9763
Resolution range [Å]	74.72–2.02 (2.29–2.02)
Completeness [%]	92.2 (71.6)
<I/σ(I)>	10.68 (1.76)
R_merge_ [%]	9.159 (34.98)
CC1/2	0.977 (0.711)
Ellipsoidal resolution (Å) (direction)	3.321 (a^∗^) 2.540 (b^∗^) 1.968 (c^∗^)
Refinement statistics	
Resolution range (Å)	74.72–2.02
Number of protein chains in a.u.	1
Included amino acids for each chain	24–198, 201–560, 564–616, 642–849, 860–1016
No. of protein atoms	7667
No. of waters	388
R_work_/R_free_ [%]	20.5/25.9
r.m.s.d. for bonds [Å]/angles [°]	0.003/0.470
Ramachandran favored/allowed [%]	96.9/3.1
Ramachandran outliers [%]	0.0
Average B–factor macromolecule [Å^2^]	37.3
Average B–factor water [Å^2^]	26.2
No of TLS groups	3

**Figure 1:**
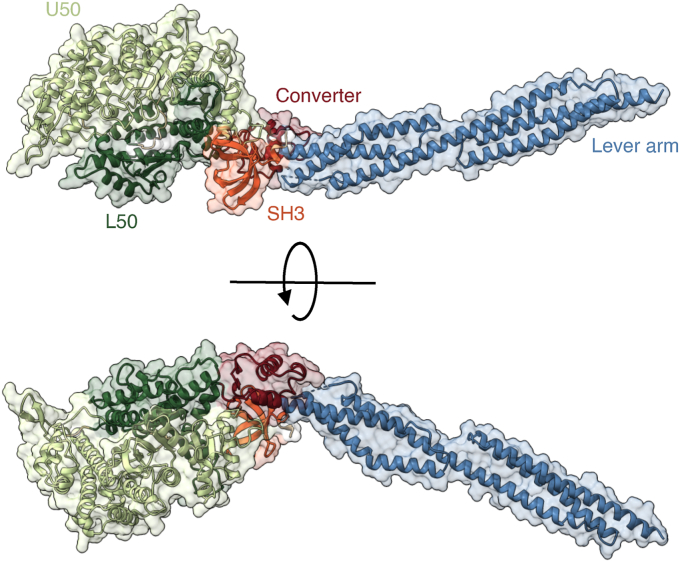
**Structure of the NM2A motor domain with an artificial lever arm.** Ribbon representation of the NM2A-2R structure with subdomains color-coded as follows: Upper 50 kDa domain (*light green*), lower 50 kDa domain (*dark green*), converter (*red*), SH3-domain (*orange*), and artificial lever (*blue*). NM2A, nonmuscle myosin 2A; SH3, Src homology 3

The final model includes the conserved myosin motor domain, which comprises the SH3 domain (SH3), upper 50 kDa domain (U50), lower 50 kDa domain (L50) and converter fused to the artificial lever arm (Fig. 1). Since NM2A crystallized in its nucleotide-free state, neither ATP nor ADP was observed in the nucleotide binding pocket.

### Comparison of NM2A, NM2B, and NM2C

**Figure 2:**
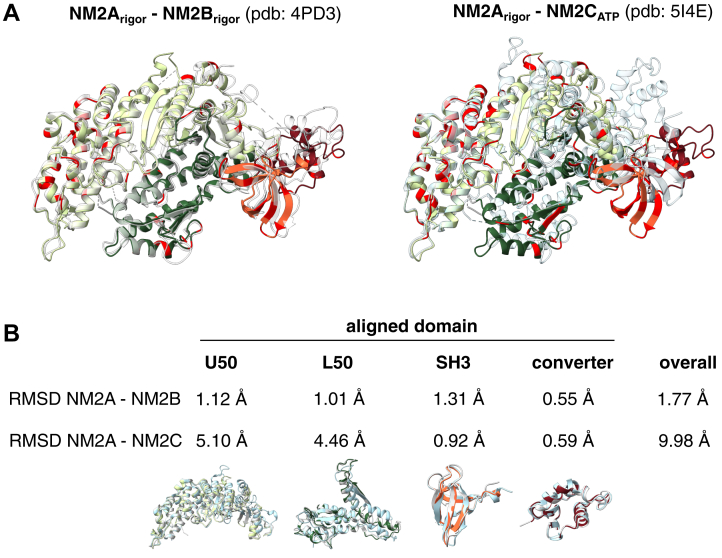
**Comparison of NM2A with NM2B and NM2C.***A*, overlay of NM2A with NM2B (PDB: 4PD3, *left*) and NM2C (PDB: 5I4E, *right*) motor domains. Differing residues are highlighted in *red*. NM2B and NM2C are shown at 50% transparency (*light gray* and *light blue*, respectively); NM2A is colored by domain. NM2A and NM2B are in the rigor state; NM2C is in the pre-powerstroke (ATP) state. *B*, RMSD values for motor domain subdomains after alignment. Below the values, overlays of the corresponding subdomains are shown using the same color scheme. NM2A, nonmuscle myosin 2A; PDB, Protein Data Bank.

To better understand the structural differences among NM2A, NM2B, and NM2C, we compared our NM2A crystal structure with previously reported structures of NM2B (Protein Data Bank (PDB): 4PD3) and NM2C (PDB: 5I4E). Since both NM2A and NM2B are in the nucleotide-free state, their comparison highlights paralogue-specific differences. In contrast, NM2C is in the ADP•P_i_ state, meaning its structural differences also reflect differences in nucleotide states, as evident from its altered overall geometry (Fig. S1). Amino acid differences between the isoforms were mapped by superimposing NM2A on NM2B and NM2C, with sequence variations highlighted in red (Fig. 2*A*). These differences are dispersed throughout the molecule without noticeable clustering (Fig. 2*A*). The root mean square deviation (RMSD) between NM2A and NM2B is 1.77 Å, whereas the RMSD between NM2A and NM2C is significantly higher at 9.98 Å (Fig. 2*B*). This large disparity is primarily due to differences in nucleotide state, which influence the opening of the cleft between the L50 and U50 domains and lead to substantial translocation of the transducer region (Fig. 2*A*, right; Fig. S1). Nonetheless, the low RMSD values for individual subdomains emphasize the structural similarity across all NM2 paralogues (Fig. 2*B*).

### Comparison of the nucleotide binding site between NM2 paralogs

**Figure 3:**
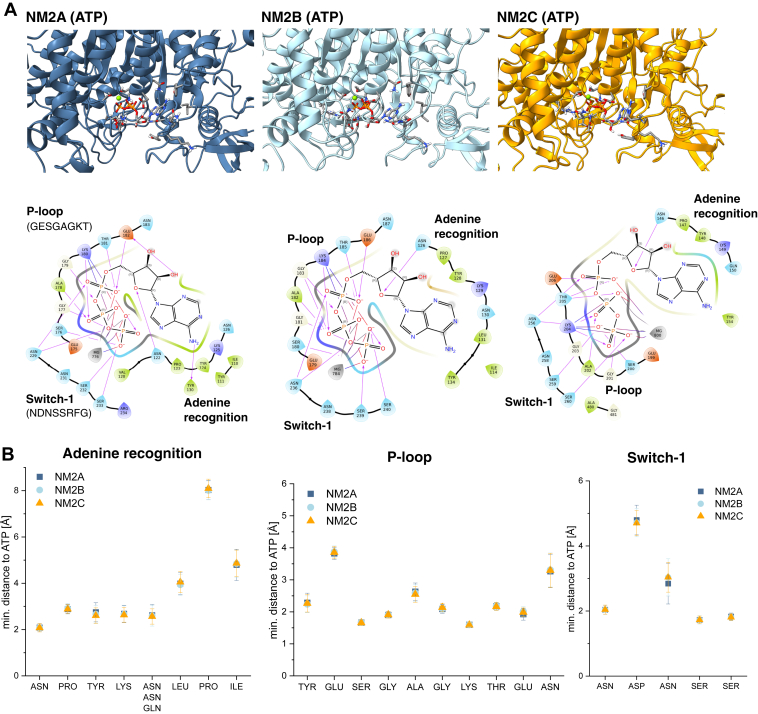
**Interaction of NM2s with ATP.***A*, ATP-binding sites shown as three-dimensional models (*upper panels*) and two-dimensional interaction diagrams (*lower panels*). *B*, minimum distances between ATP and interacting residues in the adenine recognition site, P-loop, and wwitch-1. Data are means ± S.D., averaged over the final 100 ns of each simulation and three independent replicates. No statistically significant differences were detected. NM2, nonmuscle myosin 2.

For a more detailed comparison of the NM2s, we generated homology models of NM2A and NM2B in the ATP-bound state, followed by short molecular dynamics simulations of all three NM2s and analysis of their ATP-binding sites. The 200 ns simulation time is expected to be adequate for capturing paralogue-specific effects on the ATP-binding pocket, such as side chain flipping or helix bending. However, since a full ATP-hydrolysis cycle of NM2A takes approximately 4 s, this process occurs on a much longer timescale and is therefore outside the scope of our simulations. The conserved motifs responsible for ATP binding (P-loop, switch-1, and switch-2) share identical sequences across all NM2 paralogs. The phosphate groups of ATP interact primarily with the P-loop and switch-1 (Fig. 3*A*), while switch-2 contributes a conserved glutamate that positions the catalytic water for nucleophilic attack on the γ-phosphate and stabilizes the closed nucleotide-bound conformation, linking ATP binding to conformational changes in the motor domain (38). In addition, a conserved salt bridge between Arg in switch-1 and Glu in switch-2 forms a critical element of the communication pathway linking the nucleotide- and actin-binding sites, thereby coordinating conformational changes across the motor domain (39).

To quantify paralogue-specific differences in nucleotide binding, we analyzed the minimum distances between ATP-binding residues during the second half of the simulation and compared them across paralogues. Consistent with their high sequence similarity, these distances were identical for all paralogs. Nevertheless, the analysis pinpointed key residues that maintain close proximity to ATP and exhibit minimal fluctuation during this simulation phase. Notably, Lys180 (NM2B: Lys184, NM2C: Lys204) and Ser176 (NM2B: 180, NM2C: 200) in the P-loop form stable interactions with ATP, characterized by consistently short distances and low standard deviation (Fig. 3*B*). The former interaction is particularly significant, as Lys180 is known to contribute to metaphosphate stabilization during ATP hydrolysis (40). Since no differences in ATP binding were observed, isoform specific variations are likely driven by differences in actin binding.

### Characterization of the myosin-actin interaction

**Figure 4:**
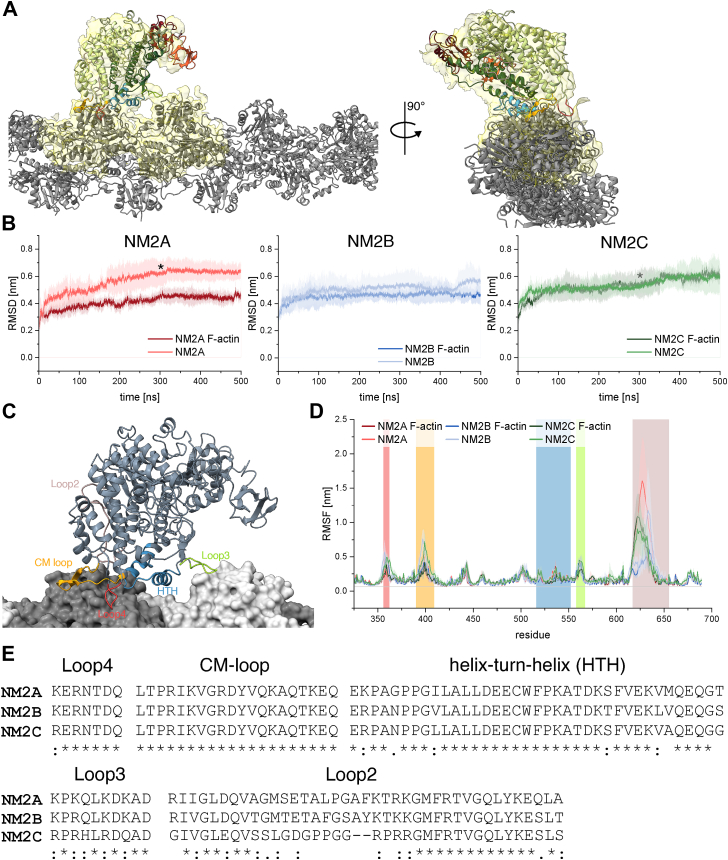
**Interaction of NM2 isoforms with the actin filament**. *A*, crystal structure of the apo-state NM2A motor domain (colored as in Fig. 1) docked into the myosin density of the actomyosin complex (*yellow*, cutoff 3σ; EMDB: EMD-8164; PDB: 5JLH). The actin filament is shown in *gray*. *B*, average RMSD ± SD of NM2A (*red*), NM2B (*blue*), and NM2C (*green*) in simulations starting from the apo motor domains positioned near the actin filament (*dark shades*) or in isolation (*light shades*). The *asterisk* (∗*) marks* termination of a shorter trajectory. *C*, starting interaction of the NM2A apo motor domain positioned near the actin filament (*gray*) with actin-interacting regions highlighted: loop4 (*red*), CM-loop (*orange*), helix–turn–helix (*blue*), loop3 (*green*), and loop2 (*brown*). *D*, RMSF of NM2A, NM2B, and NM2C in the presence and absence of actin. Motor domain regions involved in actin binding are color-coded as in panel *C*, and isoforms are colored as in panel *B*. *E*, sequence alignment of actin-interacting regions from NM2A, NM2B, and NM2C. Conservation is indicated by *asterisks* (∗) for identity, colons (:) for strong similarity, and periods (.) for weak similarity. NM2A, nonmuscle myosin 2A; PDB, Protein Data Bank; EMDB, Electron Microscopy Data Bank; RMSF, root mean square fluctuation.

Given the absence of distinct structural differences in the ATP-binding site among NM2 paralogs, actin interaction is likely the primary determinant of isoform-specific behavior. To explore the structural aspects of the myosin-actin interaction, we built an actin–myosin filament model by reconstructing missing loops and positioning the high-resolution structures of NM2A and NM2B within the myosin density of the NM2C-actin complex (Electron Microscopy Data Bank, EMDB: EMD-8164) (Fig. 4*A*). Molecular dynamics (MD) simulations were then performed to refine the models and capture the dynamic nature of the interaction. All simulations converged to an RMSD of approximately 0.6 nm, indicating structural stability with no unfolding or major rearrangements of the actin–myosin complex (Fig. 4*B*).

The interaction between NM2s and actin is primarily mediated by charged residues within myosin loop regions (41). In our model, key actin-interacting domains include loop2, loop3, loop4, the CM-loop, and the helix-turn-helix motif (Fig. 4*C*), with loop2 shifting closer to actin during the simulation. This highlights loop2 as a crucial structural element facilitating NM2-actin binding. For both NM2A and NM2B, simulations of the actin-bound complex reveal reduced loop fluctuations compared to their unbound states (Figs. 4*D* and S2), underlining the stabilizing effect of actin-binding. Among these regions, loop2 exhibits the most pronounced change in dynamics, with both NM2A and NM2B showing a significant reduction in root mean square fluctuation (RMSF) values (Figs. 4*D* and S2). NM2C does not show this decrease in fluctuation of loop2 upon actin binding, probably due to its shorter length (Figs. 4, *D* and *E* and S2).

Sequence alignment of NM2A, NM2B, and NM2C further reveals that loop2 has the highest degree of amino acid variation among actin-interacting regions (Fig. 4*E*). These sequence differences may underlie functional or mechanistic distinctions between NM2A and NM2B in actin-binding. This finding suggests that loop2 plays a pivotal role in actin interaction and may contribute to isoform-specific behavior.

Loop2 plays a multifaceted role in myosin function, with variable effects on kinetics demonstrated by chimeric and mutational studies across multiple myosin classes (41, 42, 43, 44). Mutations within loop 2 have been shown to directly affect the apparent actin affinity as well as the maximum ATPase rate. In addition, loop 2 mediates the catch-bond behavior of rigor myosin binding to actin, whereby the interaction transitions from short-lived to long-lived states under increasing force, enabling force-dependent regulation of crossbridge kinetics (45). This property is directly linked to the duty ratio and may underlie differences in duty ratio between NM2A and NM2B.

## Conclusions

We present the first high-resolution crystal structure of the NM2A motor domain, which demonstrates significant structural conservation with NM2B. Through comparative structural analysis of NM2A, NM2B, and NM2C, we identify paralogue-specific differences in amino acid residues distributed across the molecule. Molecular dynamics simulations of the ATP-bound state further reveal a high degree of similarity in the nucleotide-binding pocket across paralogues. In addition, all-atom simulations of NM2A and NM2B in both actin-bound and unbound states highlight distinct actin-binding dynamics, with loop2’s conformational flexibility emerging as key discriminators between isoforms.

Mapping of known MYH9-related disease mutation sites reveals that K373 is situated near actin-binding loops, optimally positioned to influence coupling between the actin and nucleotide binding sites. Mutations in residues N93, A95, and S96 within the L50 subdomain, along with the adjacent R702, R705, and Q706 residues in the converter (Fig. S3), are likely to disrupt communication with the neighboring SH3-like subdomain and the lever arm (22, 46, 47, 48). This is consistent with the observed 75% decrease in sliding velocity of the NM2A-R702C mutant (49).

Consistent with our model, nucleotide-dependent, actin-independent differences between NM2 isoforms are minimal. Purified isoform-enriched nonmuscle myosin II—platelet myosin (NM2A), brain myosin (NM2B), and leukocyte myosin—show similar K^+^(EDTA)- and Ca^2+^-stimulated ATPase rates (50, 51, 52), which exceed basal Mg^2+^-ATPase but differ little between isoforms. Our recombinant NM2 constructs lack the RLC binding region, yet their basal Mg^2+^-ATPase, reflecting intrinsic motor domain activity, is very low and comparable across isoforms. These observations indicate that isoform-specific functional divergence arises primarily upon actin binding, consistent with our simulations revealing distinct allosteric pathways and conformational dynamics likely to modulate ADP release and force generation.

## Limitations

Our study models ATP- and ADP•P_i_–bound states based on the NM2C-2R/ADP•VO_4_ structure, acknowledging that these biochemical states are best described as overlapping conformational ensembles rather than single structures. Accordingly, our structural models reflect these ensembles and cannot fully resolve differences specific to each nucleotide state. While our molecular dynamics simulations (up to 1.3 μs per paralogue) capture local and domain-level motions within each ensemble, they are not sufficient for exhaustive sampling of all potential conformations or slow transitions. Additionally, homology modeling and loop refinement are inherently limited in exploring the full conformational space, although subsequent MD simulations help to mitigate this constraint.

## Experimental procedures

### Protein production and purification

The motor domain of NM2A (residue 1–775) was fused to two alpha-actinin repeats of *Dictyostelium discoideum,* which function as an artificial lever arm (53). The fusion construct was ligated into a pDEST8 vector, followed by protein production in Sf9 cells and protein purification as described in (36). Briefly, the cells were lysed using sonication, and the protein was purified through Ni-NTA affinity chromatography, followed by size-exclusion chromatography. The protein was concentrated to a concentration of 10 mg/ml and stored in storage buffer (20 mM Hepes pH 7.3, 150 mM NaCl, 3 mM MgCl_2_, 1 mM β-mercaptoethanol, and 3% trehalose).

### Crystallization and structure determination

Protein crystallization was performed using 48-well MRC Maxi sitting drop plates using an Oryx8 crystallization robot (Douglas Instruments). Drops were set up by mixing 0.4 μl protein with 0.4 μl reservoir solution. The final conditions for structure determination were 16% PEG3350 and 240 mM Na-thiocyanate. Initial crystals appeared within 3 to 5 days. A cryo-protectant solution containing 25% (w/v) glycerol was used to preserve the crystals. X-ray diffraction data were collected at the Diamond Light Source on beamline I03. Diffraction data were processed automatically using the autoPROC + STARANISO pipeline provided by Diamond Light Source (54, 55, 56, 57). Molecular replacement was performed with Phaser 2.8.3 using NM2B-2R (PDB: 4PD3) as the template (58). Structure refinement was performed with *phenix.refine* (Phenix 1.20.1), alternating with manual model building in Coot 0.9.8.95 (59, 60).

### Myosin-ATP modeling

Homology models of NM2A and NM2B in the ATP state were modeled using the Prime model of the Schrödinger software suite, followed by protein preparation (61, 62). The NM2C structure with ADP•VO_4_ bound was used as a template (PBD: 5I4E). By replacing the VO_4_ group with a phosphate group, we obtained ATP-bound NM2s, as previously described (40).

### Myosin-actin complex modeling

The motor domains of NM2A (residues Ala24 to Arg775), NM2B (residues Ala32 to Arg783) and NM2C (residues Glu49 to Arg799) were used to model the respective actin-myosin complexes. Missing loops were reconstructed using MODELLER 10.5 (63). In the case of NM2B, loop2 was initially buried within the motor domain. To achieve a conformation similar to that of NM2A, the loop was manually repositioned outside the motor domain. The NM2A, NM2B, and NM2C motor domains were superimposed on NM2C in the existing actin-NM2C complex structure (PDB: 5JLH, EMDB: EMD-8164) to create the final models.

### Molecular dynamics simulations

MD simulations were set up using GROMACS 2024.3 with the NM2 structures and the NM2-actin complex structures serving as starting structures and CHARMM36 as the force field (64, 65). The NM2 or NM2-actin complex was positioned within a rectangular box, ensuring a minimum distance of 1 nm between the protein and the box walls. The box was solvated, and sodium chloride was added to achieve a final concentration of 0.15 M, neutralizing the system's overall charge. The system underwent energy minimization, followed by 100 ps of NVT and NPT equilibration, with the latter utilizing the Parrinello-Rahman barostat. The production simulation was then performed for 500 ns for the NM2-actin complex, where the positions of the backbone atoms of the actin molecules were restraint with a harmonic potential and a force constant of 1000 kJ mol^-1^ nm^-2^. The NM2 alone was simulated for 1 μs without any restraints. All simulations were carried out at 300 K, employing the V-rescale thermostat and maintaining a pressure of 1 bar using the Parrinello-Rahman barostat. Long-range electrostatic interactions were calculated using the Particle Mesh Ewald method, with a cutoff distance of 1.2 nm.

An overview of all simulations, including their individual durations, total aggregated simulation time, and the origin of their input models, is provided in Table S1.

### Structure comparison and analysis

Structures were analyzed and visualized with ChimeraX 1.9 (66, 67), which was also employed for RMSD calculations. RMSD and root mean square fluctuation values for MD trajectories were computed with GROMACS 2024.3. Specialized analysis was performed with custom Python scripts utilizing the MDAnalysis 2.7.0 library (68, 69). Distances between nucleotide-binding site residues and ATP were averaged over the final 100 ns of each independent simulation. These values were then combined across three replicates, and standard deviations were calculated to capture both intratrajectory fluctuations and inter-replicate variation. Statistical significance was evaluated using Student’s *t* test after applying block bootstrapping to mitigate false positives arising from temporal autocorrelation.

Two-dimensional ATP interaction plots were generated with the Schrödinger 2025-1 software suite (70).

## Data availability

The NM2A crystal structure has been deposited in the Protein Data Bank under the accession code 9IHL. The output models of the molecular dynamic simulations and the corresponding input settings can be accessed through Zenodo (https://doi.org/10.5281/zenodo.16893874).

## Supporting information

This article contains supporting information.

## Conflict of interest

The authors declare that they have no conflicts of interest with the contents of this article.
