# Supplementary material for: Structure of the human nonmuscle myosin 2A motor domain: Insights into isoform-specific mechanochemistry
Source: J Biol Chem. 2025 Sep 10;301(10):110691. doi: 10.1016/j.jbc.2025.110691 (PMC12546999; doi:10.1016/j.jbc.2025.110691)
Supplement: Supporting information [file mmc1.docx]

***Supporting information***

Structure of the Human Non-Muscle Myosin 2A Motor Domain: Insights into Isoform–Specific Mechanochemistry

Robin S. Heiringhoff^1,2^, Johannes N. Greve^1^, Michael Zahn^1,2,#^ and Dietmar J. Manstein^1,2,^*

From the ^1^ Institute for Biophysical Chemistry, Fritz–Hartmann–Centre for Medical Research, Hannover Medical School, 30625 Hannover, Germany, ^2^ Division for Structural Biochemistry, Hannover Medical School, 30625 Hannover, Germany

^#^ Current address: Biozentrum, Martin Luther University Halle-Wittenberg, 06120 Halle, Germany

* Corresponding author: Dietmar J. Manstein, Tel: (49) 511–5323700; Fax: (49) 511–5325966; E–mail: [manstein.dietmar@mh–hannover.de](mailto:manstein.dietmar@mh-hannover.de)

***Supplementary Figures***


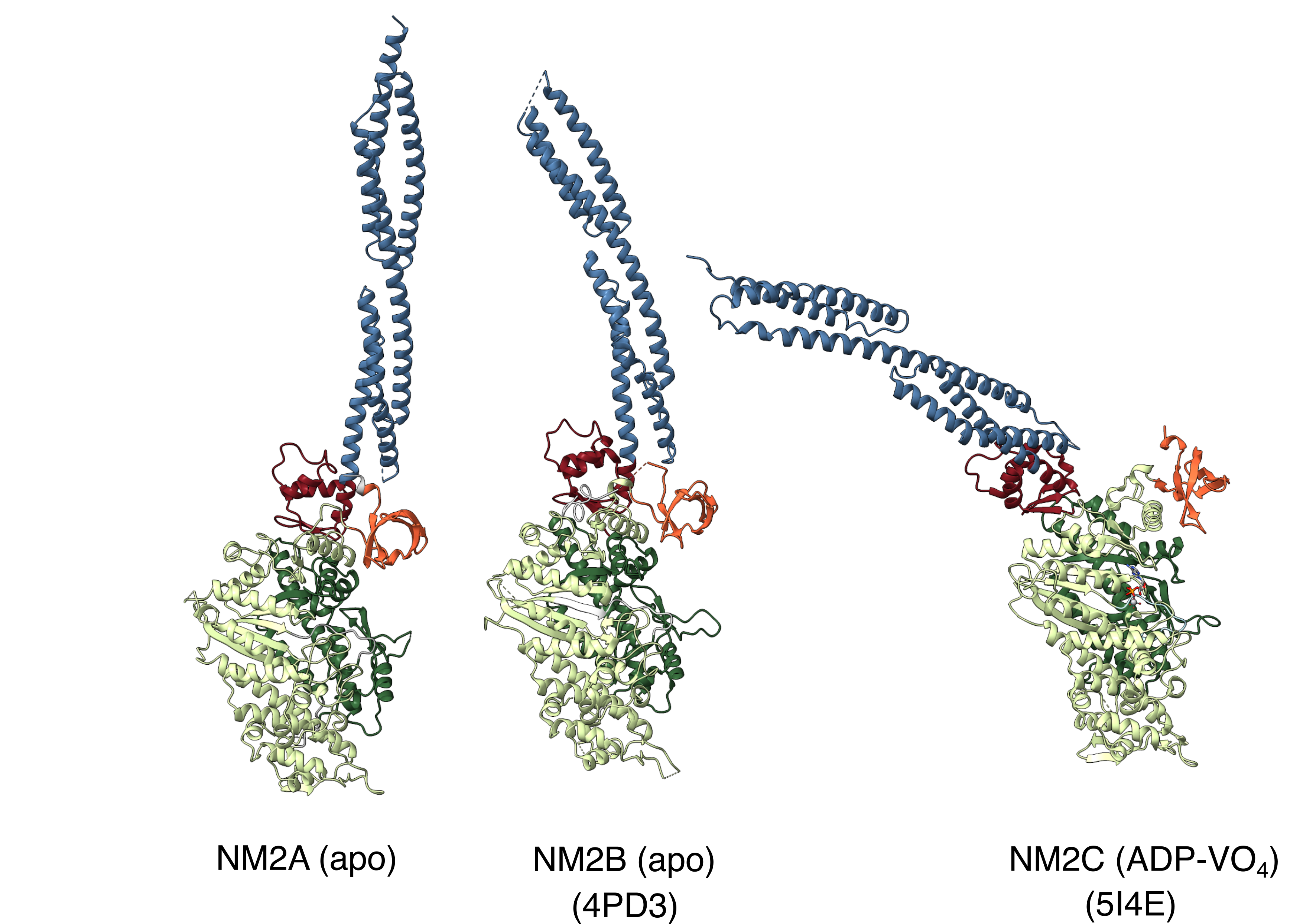


**Supplementary Figure 1.** Crystal structures of NM2A motor domain colored by subdomain. The upper 50-kDa domain is shown in light green, the lower 50-kDa domain in dark green, the converter in red, the SH3-like domain in orange, and the artificial lever in blue.









**Supplementary Figure 2.** Differences in RMSF between free and actin-bound NM2 motor domains. The plots show per-residue differences in root-mean-square fluctuation (ΔRMSF = RMSF_NM2 (with actin)_ – RMSF_NM2 (alone)_). Positive values indicate increased flexibility upon actin binding, while negative values indicate reduced flexibility.


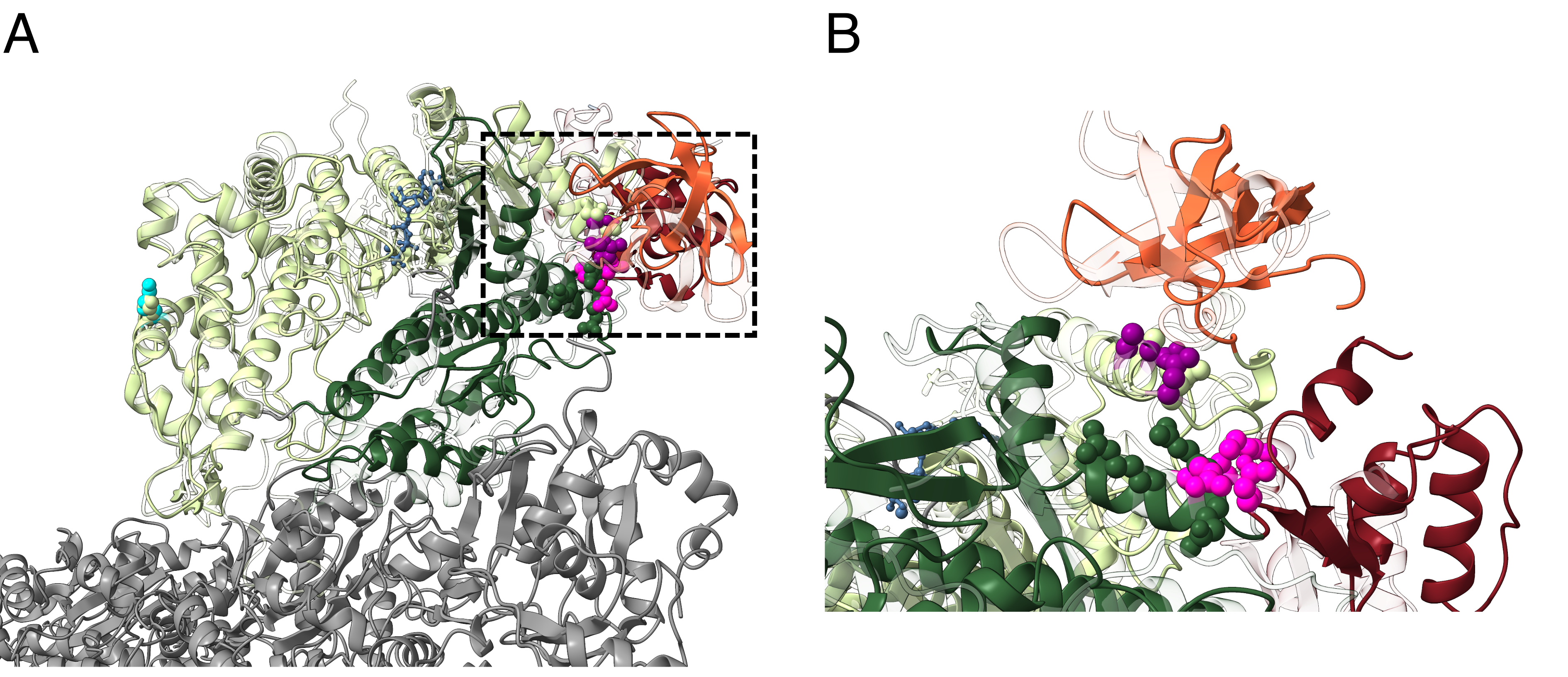


**Supplementary Figure 3. Mutation sites mapped on the NM2A motor domain.** *A*, Nucleotide-free NM2A bound to filamentous actin (grey) with color-coded subdomains, superimposed with the ATP-bound structure (U50 subdomain at 70% transparency). Mutation sites are shown as spheres; in the ATP-bound state, K373 (cyan), N93, A95, S96 (dark magenta), and R702, R705, Q706 (light magenta) are highlighted. *B*, Close-up of the SH3-like subdomain and converter region showing N93, A95, S96, R702, R705, and Q706 in both nucleotide states.

**Supplementary Table 1. Molecular dynamics simulations performed in this study.**

| **Simulations** | **time** | **runs** | **total simulation time** | **Input model**  (for all myosin models the unresolved loop regions were modeled using Modeller) |
| --- | --- | --- | --- | --- |
| **Differences in the nucleotide-binding cleft in the ATP state** | | | | |
| NM2A ATP | 200ns | 3 | 600 | Homology model of NM2A based on PDB 5I4E |
| NM2B ATP | 200ns | 3 | 600 | Homology model of NM2B based on PDB 5I4E |
| NM2C ATP | 200ns | 3 | 600 | PDB: 5I4E |
| **NM2 - actin interaction** | | | | |
| NM2A F-actin | 2x 500ns  1x 300ns | 3 | 1300 | NM2A motor domain determined in this study; modeled into EM density (EMDB: EMD-8164, containing both F-actin and myosin motor domain) |
| NM2B F-actin | 500ns | 3 | 1500 | Homology model of motor domain based on PDB: 4PD3, modeled into EM density (EMDB: EMD-8164, containing both F-actin and motor domain) |
| NM2C F-actin | 2x 500ns  1x 300ns | 3 | 1300 | Homology model of motor domain based on NM2A (this study), modeled into EM density (EMDB: EMD-8164, containing both F-actin and motor domain) |
| NM2A | 500ns | 3 | 1500 | NM2A motor domain determined in this study |
| NM2B | 500ns | 3 | 1500 | PDB: 4PD3 |
| NM2C | 500ns | 3 | 1500 | Homology model based on NM2A (this study) |
